# Cancer incidence in Thyborøn-Harboøre, Denmark: a cohort study from an industrially contaminated site

**DOI:** 10.1038/s41598-021-92446-y

**Published:** 2021-06-21

**Authors:** Elsebeth Lynge, Hans Asger Holmsgaard, Therese L. F. Holmager, Søren Lophaven

**Affiliations:** 1 Nykøbing Falster Hospital, University of Copenhagen, Ejegodvej 63, 4800 Nykøbing Falster, Denmark; 2 Ærøvej 1A, 7680 Thyborøn, Denmark; 3 Omicron, Løvparken 21, 4000 Roskilde, Denmark

**Keywords:** Cancer, Environmental social sciences

## Abstract

In a fishing community Thyborøn-Harboøre on the Danish West coast, a chemical factory polluted air, sea, and ground with > 100 xenobiotic compounds. We investigated cancer incidence in the community. A historical cohort was identified from the Central Population Register and followed for cancer incidence in the Danish Cancer Register including inhabitants from 1968–1970 at height of pollution, and newcomers in 1990–2006 after pollution control. Two fishing communities without pollution, Holmsland and Hanstholm, were referent cohorts. We calculated rate ratios (RR) and 95% confidence intervals (CI). In 1968–1970, 4914 persons lived in Thyborøn-Harboøre, and 9537 persons in Holmsland-Hanstholm. Thyborøn-Harboøre had a statistically significant excess cancer incidence compared with Holmsland-Hanstholm; RR 1.20 (95% CI 1.11–1.29) deriving from kidney and bladder cancer; stomach and lung cancer in men, and colorectal cancer in women. In 1990–2006, 2933 persons came to live in Thyborøn-Harboøre. Their cancer incidence was the same as for newcomers to Holmsland-Hanstholm; RR 1.07 (95% CI 0.88–1.30). Persons in Thyborøn-Harboøre at height of chemical pollution had a cancer risk 20% above persons living in non-polluted fishing communities with a pattern unlikely to be attributable to life style. The study suggested that chemical pollution may have affected cancer risk.

## Introduction

**Figure 1:**
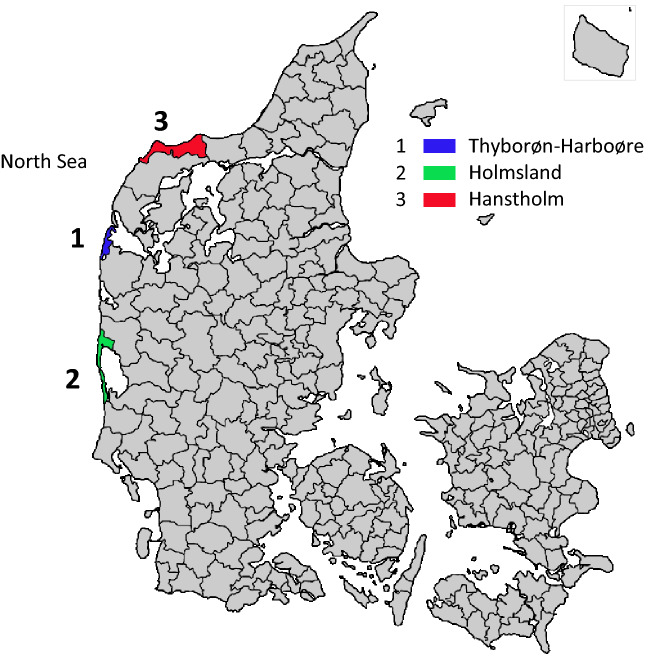
Denmark with the study area Thyborøn-Harboøre and the local control areas Holmsland and Hanstholm marked.

The chemical composition of exposures from waste dumps is often mixed and not fully known. Studies of cancer incidence in local communities in vicinity of waste dumps is an established method for evaluating the public health impact of such exposures^1,2^. In Denmark, a chemical factory producing insecticides, primarily parathion, was established on the West coast in 1953, and it is still in operation (Fig. 1). The factory was Cheminova, and in the 1950s and 1960s it caused considerable pollution problems in the local fishing community Thyborøn-Harboøre^3^. The Cheminova-case has been the most massive environmental pollution event in Denmark. A thorough investigation of the pollution in the 1980s undertaken by the local authorities^4^, led to several regulations and better control of subsequent pollution. The old pollution, however, is still of public health concern^5^.

In a previous study^6^, men ever employed at the factory for at least one year between 1953 and 1993 were followed for mortality and cancer incidence. The all-cause mortality; standardised mortality ratio (SMR) 0.96 (95% confidence interval (CI) 0.82–1.12), and the overall cancer incidence; standardised incidence ratio (SIR) 0.98 (95% CI 0.79–1.21), did not differ from the level expected for Danish men. However, in 2015 one of the authors (HAH), after having worked for many years as a general practitioner in the area and having observed a seemingly high number of deaths due to cancer in the local population, asked the health authorities to investigate the cancer incidence in the area. The request was circulated among a number of governmental agencies, but no investigation was undertaken. Parathion was classified as a possible human carcinogen (Group 2B) by the International Agency for Research on Cancer (IARC) in 2015^7^, and although the environmental pollution around Cheminova derived mostly from the waste products, the IARC evaluation highlighted the interest in cancer incidence in Thyborøn-Harboøre. On this background the present study was undertaken.

We evaluated the cancer incidence among persons living in Thyborøn-Harboøre, and compared with cancer incidence data from two fishing communities without chemical factories. We identified two cohorts; persons living in Thyborøn-Harboøre in 1968–1970 during the height of the chemical pollution, and newcomers to Thyborøn-Harboøre in 1990–2006 after pollution control had been implemented. This allowed us to determine whether the chemical pollution in the 1950s and 1960s left marks on the health of the people living in the area at that time, and whether the environmental pollution from this era affected the newcomers.

## Methods

### Pollution

**Figure 2:**
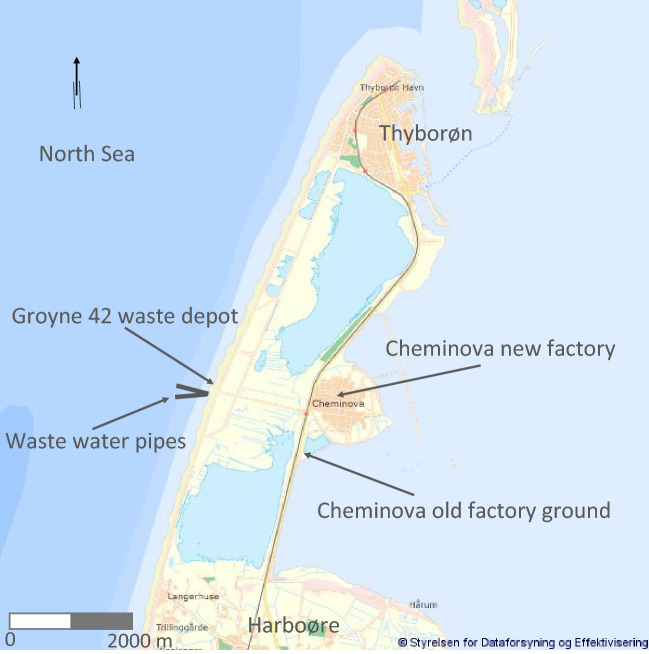
Detailed map of Thyborøn-Harboøre. Background map used with permission from The Danish Agency for Data Supply and Efficiency and adapted with details from^4^.

The pollution from the factory came from different sources; from the old and the new factory grounds (Fig. 2); from waste water; from waste dumps, in particular at Groyne 42 at the West coast; and from release of chemicals into the air. Concern about the pollution started in the 1950s when dead fish and birds were observed^3^, and intensified in 1964 when large number of lobsters died along the West coast and in the bay on the opposite side of the peninsula. Investigations in the 1980s found waste water from the factory to include more than 100 xenobiotic compounds. The air pollution from the factory came in particular from release of sulphur dioxide and solvents. In the waste dumps both sulphur, phosphor^4^, and mercury have been found^5^. Since 1981, health concerns about the pollution led to interventions to clean waste water and air before its release, and to recovery and sealing off of some of the waste dumps. The pollution around the factory is, however, still of concern, as the area is not cleaned completely, and there are still traces of the waste in the bay water but within limit values^8–10^.

### Population

The study was undertaken as a historical cohort study. From the Danish Central Population Register we identified all persons who ever had an address in Thyborøn-Harboøre between 1968 and 2006. As the old municipality codes ceased to exist by 1 January 2007, we did not include persons born in/moving to the areas from 1 January 2007 onwards. Thyborøn has one of five large fishing harbours in Denmark. Harboøre is geographically adjacent to Thyborøn, and the two communities share water supply. As a comparison group, we used all persons ever with an address in Holmsland and Hanstholm between 1968 and 2006, and not belonging to the study group. Like Thyborøn-Harboøre, Holmsland and Hanstholm are located at the Danish West coast but so far away that they cannot have been affected by the water contamination from Thyborøn (Fig. 1), and both places have large fishing harbours. These areas were chosen as a geographical comparison group to represent fishing communities like Thyborøn-Harboøre, but without pollution from chemical factories.

From the Thyborøn-Harboøre population, we formed two cohorts. First, persons living in Thyborøn-Harboøre at the height of the chemical pollution in 1968–1970. Second, newcomers to Thyborøn-Harboøre in 1990–2006 after the release of chemicals to the environment came under control from the end of the 1980s.

### Follow-up for emigration and death

In the 1968–1970 Thyborøn-Harboøre cohort, a person entered the study group on the first date with an address in Thyborøn-Harboøre, between 2 April 1968 and 31 December 1970. A person left the study group on date of first emigration from Denmark, death, or end of follow up on 31 December 2017, whichever came first. The same restrictions on dates were used for the geographical comparison group, with the exception that a person could not be included in the comparison group if he/she had previously belonged to the study group. For each person, person-years at risk were accumulated from date of entry to date of exit. For the 1968–1970 cohort, length of stay in Thyborøn-Harboiøre was estimated as time from the start of the factory 1 January 1953 or from date of birth, whichever came latest, until first registered date of leaving the area from 1968 onwards up intil 31 December 2017. The same definition was used for estimation of length of stay in Holmsland-Hanstholm.

In the 1990–2006 Thyborøn-Harboøre cohort, a given person entered the study group on 1 January 1990 or later up until 31 December 2006, provided he/she had not lived in Thyborøn-Harboøre before 1 January 1990. Definition of exit, geographical comparison group, and accumulation of person years followed the principles used for the 1968–1970 cohort. For the 1990–2006 cohort, length of stay in Thyborøn-Harboøre was calculated as time from first registered date in Thyborøn-Harboøre from 1 January 1990 onwards, until first registered date of leaving the area up until 31 December 2017. The same definition was used for calculation of length of stay in Holmsland-Hanstholm.

Data on births, emigrations, and deaths were retrieved from the Central Population Register (CPR). This was the case also for dates on changes of addresses from 1 January 1971 to 31 December 2006. From 2 April 1968 to 31 December 1970, dates on changes of addresses were estimated from archived, cross-sectional CPR-versions available from 2 April 1968, 12 June 1969, and 13 April 1970. For a person not present in the area in version x but in version x + 1, we assumed that the person moved to the area on the mid-day between the two CPR-versions.

### Cancer incidence

In Denmark, nationwide cancer incidence data are available from 1943 onwards. The study populations and the geographical comparison groups were linked with the Danish Cancer Register to identify incident cancer cases within the individual risk periods. Cancer cases diagnosed from 1968 to 1977 were coded according to a modified version of the International Classification of Diseases version 7 (ICD7), and cancer cases diagnosed 1978 to 31 December 2017 were coded according to ICD10. Both ICD7 and ICD10 codes had been converted to NORDCAN codes^11^.

### Statistical analysis

Both for the 1968–1970 and the 1990–2006 cohorts, rate ratios (RR) between the study groups and the geographical comparison groups were calculated overall as well as for men and women separately, using Poisson regression and controlling for running age in 5-years age group, 5-year calendar period of the follow-up time, and for the overall analysis also for sex. The 95% CIs were calculated as Wald confidence intervals. Supplementary analyses stratified the population by broad age-groups; 0–39, 40–59, and 60 + years, and with comparison of the 1990–2006 Thyborøn-Harboøre cohort with that from 1968–1970. In addition, we calculated the absolute number of extra cancer cases per 100,000 person years as [((observed number – (observed number/RR))/person years) × 100,000]. As we are not permitted to publish cancer incidence based on less than 5 observed cases, some RR values were omitted from the tables. SAS 9.4 was used for the analysis.

## Results

In Thyborøn-Harboøre, 4914 persons living in the area in 1968–1970 contributed 194,072 person years and 1108 cancer cases. The estimated mean time of having lived in Thyborøn-Harboøre was 26.3 years. Follow-up started in 1968, and a person was on average followed up for 39.5 years, and 41% of the person years were accumulated in the age-group 0–39 years, 36% in age 40–59 years, and 24% in age 60 + years. In Holmsland-Hanstholm, 9537 persons living in the area in 1968–1970 contributed 367,492 person years and 1869 cancer cases. The estimated mean time of having lived in Holmsland-Hanstholm was 20.8 years. Follow-up started in 1968, and a person was on average followed up for 38.5 years, and 40% of the person years were accumulated at age 0–39 years, 35% at age 40–59 years, and 25% at age 60 + years (Table 1).

**Table 1:** Thyborøn-Harboøre and Holmsland-Hanstholm, Denmark. Number of persons, follow-up, person-years, and cancer cases 1968–1970 and 1968–2006.

Area and period	Number of	Total		Men		Women	
**Thyborøn-Harboøre**							
Old cohort: 1968–1970	Persons	4914		2595		2319	
	Mean years of stay	26.3		26.0		26.7	
	Follow-up mean years	39.5		38.3		40.8	
	Person years/Cancers	194,072 (100%)	1108 (100%)	99,490 (100%)	599 (100%)	94,582 (100%)	509 (100%)
0–39 years	Person years/Cancers	79,167 (41%)	37 (3%)	41,270 (42%)	16 (3%)	37,897 (40%)	21 (4%)
40–59 years	Person years/Cancers	68,918 (36%)	300 (27%)	35,923 (36%)	139 (23%)	32,995 (35%)	161 (32%)
60 + years	Person year/Cancers	45,987 (24%)	771 (70%)	22,297 (22%)	444 (74%)	23,690 (25%)	327 (64%)
New cohort: 1990–2006	Persons	2933		1594		1339	
	Mean years of stay	6.8		6.6		7.1	
	Follow-up mean years	18.5		18.4		18.6	
	Person years/Cancers	54,287 (100%)	126 (100%)	29,382 (100%)	64 (100%)	24,905 (100%)	62 (100%)
0–39 years	Person years/Cancers	40,807 (75%)	16 (13%)	22,018 (75%)	6 (9%)	18,789 (75% )	10 (16%)
40–59 years	Person years/Cancers	9689 (18%)	39 (31%)	5304 (18%)	16 (25%)	4385 (18%)	23 (37%)
60 + years	Person years/Cancers	3791 (7%)	71 (56%)	2060 (7%)	42 (66%)	1731 (7%)	29 (47%)
**Holmsland-Hanstholm**							
Old cohort: 1968–1970	Persons	9537		5001		4536	
	Mean years of stay	20.8		21.0		20.6	
	Follow-up mean years	38.5		37.4		39.8	
	Person years/Cancers	367,492 (100%)	1869 (100%)	187,098 (100%)	1022 (100%)	180,394 (100%)	847 (100%)
0–39 years	Person years/Cancers	146,790 (40%)	70 (4%)	75,411 (40%)	28 (3%)	71,379 (40%)	42 (5%)
40–59 years	Person years/Cancers	127,443 (35%)	455 (24%)	66,032 (35%)	190 (19%)	61,411 (34%)	265 (31%)
60 + years	Person years/Cancers	93,259 (25%)	1344 (72%)	45,655 (24%)	804 (79%)	47,604 (26%)	540 (64%)
New cohort: 1990–2006	Persons	10,670		5615		5055	
	Mean years of stay	5.3		5.0		5.7	
	Follow-up mean years	17.5		17.1		17.9	
	Person years/Cancers	186,222 (100%)	553 (100%)	95,888 (100%)	307 (100%)	90,334 (100%)	246 (100%)
0–39 years	Person years/Cancers	124,672 (67%)	63 (11%)	63,002 (66%)	27 (9%)	61,670 (68%)	36 (15%)
40–59 years	Person years/Cancers	42,158 (23%)	190 (34%)	22,768 (34%)	88 (29%)	19,390 (21%)	102 (41%)
60 + years	Person years/Cancers	19,392 (10%)	300 (54%)	10,118 (11%)	192 (63%)	9274 (10%)	108 (44%)

Persons living in Thyborøn-Harboøre in 1968–1970 had an elevated risk of cancer incidence compared with all persons living in Holmsland-Hanstholm at the same time; RR 1.20 (95% CI 1.11–1.29) and for both men; RR 1.19 (95% CI 1.08–1.32) and women; RR 1.20 (95% CI 1.08–1.34) (Table 2). This represented 95 extra cancer cases per 100,000 person years. For men, the excess risks were seen for cancers of the stomach; RR 1.81 (95% CI 1.12–2.92), lung; RR 1.52 (95% CI 1.19–1.93), kidney; RR 1.92 (95% CI 1.13–3.25); and bladder and urinary tract; RR 1.76 (9% CI 1.31–2.36). For women, excess risks were seen for cancers of the colon/rectum; RR 1.65 (95% CI 1.26–2.15); kidney; RR 2.48 (95% CI 1.07–5.75), and bladder and urinary tract; RR 1.98 (95% CI 1.14–3.46). In the total cohort of men and women, the elevated risks of kidney and bladder cancer together represented 35 extra cancer cases per 100,000 person years.

**Table 2:** Cancer incidence in Thyborøn-Harboøre in 1968–1970 compared with cancer incidence in geographical control group.

Cancer site	Total			Men			Women		
	N_exp+_	N_exp-_	RR (95% CI)	N_exp+_	N_exp-_	RR (95% CI)	N_exp+_	N_exp-_	RR (95% CI)
All cancer	1108	1869	1.20 (1.11, 1.29)	599	1022	1.19 (1.08, 1.32)	509	847	1.20 (1.08, 1.34)
All cancer, excl. other skin	1073	1789	1.21 (1.12, 1.31)	576	969	1.21 (1.09, 1.34)	497	820	1.21 (1.08, 1.35)
Lip	6	25	0.50 (0.20, 1.22)		21				
Oral cavity	7	18	0.75 (0.31, 1.79)		14				
Oropharynx	8	8	1.94 (0.73, 5.18)	8	6	2.58 (0.89, 7.45)			
Oesophagus	19	34	1.09 (0.62, 1.91)	15	30	0.98 (0.53, 1.82)			
Stomach	40	51	1.71 (1.13, 2.59)	31	37	1.81 (1.12, 2.92)	9	14	1.42 (0.61, 3.31)
Small bowel	6								
Colon and rectum	187	279	1.34 (1.12, 1.62)	88	159	1.11 (0.85, 1.44)	99	120	1.65 (1.26, 2.15)
Colon	112	179	1.25 (0.99, 1.59)	48	98	0.99 (0.70, 1.39)	64	81	1.57 (1.13, 2.19)
Rectum	80	104	1.53 (1.14, 2.05)	41	62	1.32 (0.89, 1.96)	39	42	1.83 (1.18, 2.84)
Primary liver	10	22	0.90 (0.43, 1.90)		16		6	6	2.08 (0.67, 6.47)
Pancreas	30	50	1.21 (0.77, 1.91)	19	27	1.46 (0.81, 2.62)	11	23	0.94 (0.46, 1.93)
Larynx	20	20	1.95 (1.05, 3.63)	17	19	1.76 (0.91, 3.39)			
Lung	181	260	1.36 (1.12, 1.64)	119	156	1.52 (1.19, 1.93)	62	104	1.14 (0.83, 1.56)
Breast	142	231	1.19 (0.97, 1.47)				139	230	1.17 (0.95, 1.45)
Cervix uteri	17	34	0.95 (0.53, 1.70)				17	34	0.95 (0.53, 1.70)
Corpus uterus	29	41	1.40 (0.87, 2.26)				29	41	1.40 (0.87, 2.26)
Ovary	26	38	1.33 (0.81, 2.19)				26	38	1.33 (0.81, 2.19)
Prostate	107	216	0.99 (0.78, 1.25)	107	216	0.99 (0.78, 1.25)			
Testis	9	14	1.19 (0.52, 2.76)	9	14	1.19 (0.52, 2.76)			
Kidney	39	38	2.07 (1.32, 3.24)	27	28	1.92 (1.13, 3.25)	12	10	2.48 (1.07, 5.75)
Bladder and urinary tract	107	121	1.80 (1.39, 2.34)	82	96	1.76 (1.31, 2.36)	25	25	1.98 (1.14, 3.46)
Malignant melanoma	43	61	1.35 (0.91, 1.99)	15	17	1.68 (0.84, 3.37)	28	44	1.22 (0.76, 1.96)
Other skin	56	109	1.06 (0.77, 1.46)	37	71	1.08 (0.73, 1.61)	19	38	1.02 (0.59, 1.78)
Brain and CNS	32	59	1.03 (0.67, 1.59)	13	30	0.83 (0.43, 1.59)	19	29	1.24 (0.70, 2.22)
Non-Hodgkin lymphoma	32	58	1.08 (0.70, 1.66)	16	30	1.04 (0.56, 1.90)	16	28	1.12 (0.60, 2.07)
Myelomatosis	9	23	0.78 (0.36, 1.68)	6	13	0.93 (0.35, 2.44)	3	10	
Leukemia	30	46	1.32 (0.83, 2.10)	22	29	1.55 (0.89, 2.70)	8	17	0.94 (0.41, 2.19)
Other specified	6	7	1.71 (0.57, 5.11)						
Unknown and unspecified	52	95	1.15 (0.82, 1.61)	27	54	1.07 (0.67, 1.70)	25	41	1.25 (0.76, 2.06)

Number of cancer cases in Thyborøn-Harboøre (N_exp+_), number of cancer cases in Holmsland-Hanstholm (N_exp-_).rate ratio (RR), and 95% confidence interval (CI). RR adjusted for running age in 5-years age group, 5-year calendar period of follow-up time, and for total also for sex. Blank cell means cancer site not relevant in that sex, or less than 5 observed cases.

When the comparison of cancer incidence between the two areas was stratified by age, the cancer incidence in the Thyborøn-Harboøre cohort in the age-group 0–39 years was in line with that from Holmsland-Hanstholm, RR 0.99 (95% CI 0.66–1.47), while the excess cancer incidence in Thyborøn-Harboøre was seen both in the age-group 40–59 years and 60 + years, the RRs being 1.23 (95% CI 1.07–1.43) and 1.19 (96% CI 1.09–1.30), respectively.

The 2933 persons, who came to live in Thyborøn-Harboøre in 1990–2006, contributed 54,287 person years and 126 cancer cases. The mean time of having lived in Thyborøn-Harboøre was 6.8 years. A person in this group was on average followed up for 18.5 years, and 75% of the person years were accumulated at age 0–39, 18% at age 40–59 years, and 7% at age 60 + years. In the period 1990–2006, 10,670 persons who came to live in Holmsland-Hanstholm contributing 186,222 person years and 553 cancer cases. The mean time of having lived in Holmsland-Hanstholm was 5.3 years. A person was on average followed up for 17.5 years, and 67% of the persons years were accumulated at age 0–39 years, 23% at age 40–59 years, and 10% at age 60 + years (Table 1). The overall cancer incidence of newcomers to Thyborøn-Harboøre in 1990–2006 was in line with that of newcomers to Holmsland-Hanstholm in the same period; RR 1.07 (95% CI 0.88–1.30) (Table 3). None of the excess cancer risks for specific cancer sites seen in the 1968–1970 Thyborøn-Harboøre cohort as compared with the Holmsland-Hanstholm cohort were seen when newcomers to the two areas in 1990–2006 were compared. Newcoming women to Thyborøn-Harboøre had an excess lung cancer risk; RR 2.42 (95% CI 1.25–4.68), not seen in newcoming men; RR 1.05 (95% CI 0.53–2.09).

**Table 3:** Cancer incidence in Thyborøn-Harboøre in 1990–2006 compared with cancer incidence in geographical control group.

Cancer site	Total			Men			Women		
	N_exp+_	N_exp-_	RR (95% CI)	N_exp+_	N_exp-_	RR (95% CI)	N_exp+_	N_exp-_	RR (95% CI)
All cancer	126	553	1.07 (0.88, 1.30)	64	307	0.94 (0.72, 1.23)	62	246	1.23 (0.93, 1.63)
All cancer, excl. other skin	122	534	1.07 (0.88, 1.30)	62	297	0.94 (0.71, 1.23)	60	237	1.23 (0.93, 1.64)
Colon and rectum	14	60	1.19 (0.66, 2.13)	8	34	1.23 (0.57, 2.67)	6	26	1.16 (0.48, 2.83)
Colon	7	32	1.13 (0.50, 2.58)		19			13	
Rectum	7	29	1.16 (0.51, 2.66)	5	16	1.56 (0.57, 4.30)		13	
Pancreas	5	20	1.23 (0.46, 3.29)		12			8	
Lung	23	73	1.55 (0.97, 2.48)	10	45	1.05 (0.53, 2.09)	13	28	2.42 (1.25, 4.68)
Breast	16	56	1.38 (0.79, 2.41)				16	56	1.38 (0.79, 2.41)
Prostate	10	64	0.79 (0.40, 1.54)	10	64	0.79 (0.40, 1.54)			
Kidney	5	15	1.44 (0.52, 3.99)		11			4	
Bladder and urinary tract	11	40	1.40 (0.72, 2.74)	7	34	1.05 (0.46, 2.38)		6	
Other skin	5	22	1.24 (0.47, 3.27)		13			9	
Brain and CNS	7	27	1.04 (0.45, 2.39)		12			15	

Number of cancer cases in Thyborøn-Harboøre (N_exp+_), number of cancer cases in Holmsland-Hanstholm (N_exp-_), rate ratio (RR), and 95% confidence interval (CI). RR adjusted for running age in 5-years age group, 5-year calendar period of follow-up time, and for total also for sex. Only cancer sites with 5 + cancer cases. Blank cell means cancer site not relevant in that sex, or less than 5 observed cases.

An internal comparison between the newcomers to Thyborøn-Harboøre in 1990–2006 with those who lived there in 1968–1970 showed the same overall cancer incidence; i.e. RR 1.00 (95%CI 0.82–1.21). This result was expected because 75% of the person-years in the newcomer cohort were accumulated in the age-group 0–39 years, and in this age-group persons from both the old and the new Thyborøn-Harboøre cohorts had the same cancer incidence as persons from Holmsland-Hanstholm.

## Discussion

Persons living in Thyborøn-Harboøre at the height of the environmental pollution in the late 1960s had a 20% higher cancer incidence than persons living in similar fishing communities without environmental pollution from chemical factories. Risks for kidney and bladder cancer were roughly doubled for both men and women. In addition, men experienced an 81% elevated risk of stomach cancer, and a 52% elevated risk of lung cancer, while women had a 65% elevated risk of colorectal cancer. Below the age of 40 years, persons in Thyborøn-Harboøre experienced the same cancer incidence as persons in the other fishing communities. For cancers overall, the elevated risks represented 95 extra incident cancer cases per 100,000 person years, with 35 of these cases coming from kidney and bladder cancer. The elevated risk was thus a considerable disease burden seen in the perspective of a rare cancer being defined as an incidence of less than 6 cases per 100,000 person years^12^.

Newcomers to Thyborøn-Harboøre from 1990 after pollution control was implemented had the same cancer incidence as newcomers to the similar fishing communities in this period. However, both of these cohorts were still fairly young with 75% of the person years accumulated below the age of 40 years.

Excess cancer risks can derive from genetic predispositions, personal habits like smoking, or from environmental exposures. A genetic predisposition was unlikely to explain the observed excess cancer risks for persons living in Thyborøn-Harboøre in 1968–1970, as the Danish population is genetically very homogenous^13^.

Tobacco smoking is a strong risk factor for cancers of the kidney, bladder and lung^14^. One could hypothesize therefore that the excess risks of these cancers in Thyborøn-Harboøre derived from excessive smoking, but if this should be the sole explanation for the excess cancer risks, the lung cancer risk should be more elevated than those for kidney and bladder cancer, and one would expect to see elevated risks also for other tobacco-related cancers. This was not the case. For inhabitants from the early period, men had a 50% excess risk of lung cancer, but 80–90% excess risks of kidney and bladder cancer, and women had no excess risk of lung cancer but 100–150% excess risks of kidney and bladder cancer. Tobacco smoking was therefore unlikely to explain the observed cancer pattern, and we therefore refrained from a more detailed confounder control for smoking^15^.

Dietary factors are strong risk factors for stomach and colorectal cancer^16^. Fish was a main component of diet in fishing communities back in time. The diet in the Danish fishing communities about fifty years ago is therefore likely to have differed from the national average, but it is unlikely that differences in dietary patterns between the fishing communities at the Danish West coast could explain the observed differences in their risks of digestive tract cancers. The risk of stomach cancer was increased in men, but not in women. This disease is associated with helicobacter pylori infection and it is more frequent in people with limited access to hygiene^17^. One might hypothesize therefore that the excess risk of stomach cancer in men was associated with long stays at sea, but this would have been part of life also for men in the other fishing communities we used for comparison. The risk of colorectal cancer was increased in women, but not in men. Abdominal adiposity is a known risk factor for colorectal cancer, but actually stronger so for men than for women^18^, and it is hard to imagine that overweight has differed between women in the fishing communities.

In the previous study of cancer incidence in men employed for at least one year between 1953 and 1993 at Cheminova, the overall cancer incidence was the same as in the Danish population^6^. This is in line with findings from other studies of workers employed in Danish factories located in provincial or rural settings^19^. Both the overall lower cancer incidence in rural areas^20^, and the healthy worker effect^21^ should therefore be considered in the interpretation of these data. In total, there were 84 cancers in men in the Cheminova study; 6 stomach cancers; SIR 1.84 (95% CI 0.67–3.99); 20 lung cancers; SIR 1.29 (95% CI 0.78–1.99); and 10 bladder cancers; SIR 1.40 (95% CI 0.67–2.58). With the limitation caused by small numbers, the factory data were thus compatible with the community data.

The identification of specific cancer excess risks among persons living in Thyborøn-Harboøre in the late 1960s compared with persons living in other West coast fishing communities could indicate that environmental exposures played a role, especially for kidney and bladder cancers, which were in excess in both men and women. The excess risk of stomach cancer in men and of colorectal cancer in women may have other explanations. It is, however, also possible that environmental exposures could have reached men and women differently, as they might have had different tasks in the community. The factory study showed for instance that 1275 men had been employed at Cheminova for at least one year between 1953 to 1993, but only 192 women^6^. The environmental exposures came from polluted air; from contamination of sea water by waste water; and from dissemination of pollutants from stored waste. The chemical composition of the pollution was complicated, including more than 100 xenobiotic compounds in the waste water. It was therefore beyond the capacity of the present study to identify specific agents, if any, that might have been involved.

The elevated cancer incidence in persons living in Thyborøn-Harboøre in 1968–1970 was not seen under the age of 40 years. This might explain why the cancer incidence was not elevated among the newcomers to Thyborøn-Harboøre in 1990–2006, because 75% of their accumulated person years were under the age of 40 years. This observation illustrates the difficulties in evaluating the potential impact of pollution control, because there is a long latency period between exposure and eventual cancer occurrence.

It was a strength of the study that we could identify and follow all persons ever living in the Thyborøn-Harboøre from 1968 to 2006. We identified a comparison group from similar fishing communities without the environmental exposures in Thyborøn-Harboøre. For the entire period, individual population records could be linked with individual cancer register records.

Our population data were left censored from the start of CPR on 2 April 1968, and we were therefore not able to identify all inhabitants from Thyborøn-Harboøre from the start of Cheminova in 1953. We had to estimate mean time of having lived in Thyborøn-Harboøre. Analysis by latency since start of exposure was therefore not possible. The inhabitants we could identify from 1968–1970 were, however, expected to be fairly representative of the population living in the area at the height of the environmental pollution.

We analysed the cancer incidence for a number of cancer sites and for both men and women. Some excess risks could, therefore, be generated by chance, but so could deficit risks. It was noteworthy that not a single RR was statistically significantly decreased. Also, we could not show risk estimates based on less than 5 observed cases and some of these could have been excesses or deficits.

The study was limited by the fact that we could not control at the individual level for a potential impact of tobacco smoking and dietary factors, but the observed cancer pattern did not appear to indicate that the excess cancer risks could be attributable to personal life style. It was a limitation that we could not point to possible associations between specific exposures and cancer risks. This means that we could not evaluate to what extent our findings might be supported by the literature on chemical carcinogenesis.

Although pollution control was implemented from the late 1980s, we were not able to assess whether these efforts had affected the cancer pattern, as the newcomers to Thyborøn-Harboøre were still too young for an eventual effect of the pollution to have materialize.

The environmental pollution of Thyborøn-Harboøre has been an issue of public debate in Denmark ever since the start of the chemical factory in 1953. The wide range of xenobiotic chemicals found in the waste water from the factory has been a complicating factor^4^. Research-wise it has not been possible to pinpoint the specific susceptible association(s) between chemical(s) and health risk(s) to look for. The construction of the exposed cohort of inhabitants from Thyborøn-Harboøre was furthermore a cumbersome job, which we could undertake only because we developed the methodology for other studies^22^. It is on this background not surprising that the present study was the first one to follow up on the decades-long, local concern about the possible impact of the pollution on the health of inhabitants. However, one can argue also that with the long latency time between a possible carcinogenic exposure and development of cancer, only studies with a long-term follow-up period make sense. The fact that there were 95 extra cancer cases per 100,000 person years among people living in Thyborøn-Harboøre at the height of the pollution indicates that the local concern was justified.

After years of negotiation, the Danish parliament in the fiscal law for 2021 reserved 70 million Euro for the cleaning up of the Thyborøn-Harboøre pollution; both the old factory ground and the waste dumps at groyne 42^23^. Chemical analysis of the excavated waste might provide the possibility to establish a link between the pollution and the cancer risks observed in the present study. It should though be taken into account that part of the waste can have been degraded or washed out since the height of the pollution in the 1950s and 1960s. We found no excess cancer risk in people who moved to/were born in Thyborøn-Harboøre after 1990, but these people were still young, and if they have an elevated cancer risk this will materialize only when they age. The cleaning up of the pollution in the area will relieve future generations in the area of this possible risk.

## Conclusions

We observed a 20% elevated cancer risk for persons living in Thyborøn-Harboøre in the late 1960s in comparison with persons living in other fishing communities at the West coast of Denmark. The excess risk derived from kidney and bladder cancer in both men and women; and from stomach and lung cancer in men, and colorectal cancer in women. These results suggested that environmental pollution might have an impact on this cancer pattern, but it was beyond the capacity of the present study to investigate potential specific associations.

### Ethics approval and consent to participate

In Denmark, approval for use of data (REG-108–2018 in Region Zealand) serves as ethical clearance for register-based research. No contact was made to patients, their relatives, and/or treating physicians.

### Consent for publication

Not applicable.

## Supplementary Information

Supplementary Table.

## Data Availability

Data were stored at Statistics Denmark, and accessed via the research service facility there. Access to data can be obtained according to the Danish data protection legislation.

## Abbreviations

CI: Confidence interval
RR: Rate ratio
