# Supplementary material for: Cancer incidence in Thyborøn-Harboøre, Denmark: a cohort study from an industrially contaminated site
Source: Sci Rep. 2021 Jun 21;11:13006. doi: 10.1038/s41598-021-92446-y (PMC8217208; doi:10.1038/s41598-021-92446-y)
Supplement: Supplementary file 1 — Supplementary Table. [file 41598_2021_92446_MOESM1_ESM.pdf]

Nykøbing Falster Hospital

University of Copenhagen

15 December 2020

Revised 29 April 2021

# Cancer incidence in Thyborøn-Harboøre, Denmark

## A cohort study from an industrially contaminated site

Elsebeth Lynge[1], Hans Asger Holmsgaard[2], Therese LF Holmager[3], Søren Lophaven[4]

Supplementary Tables

SUPPLEMENTARY TABLES

Supplementary Table 1. Cancer incidence for persons in Thyborøn-Harboøre in 1968-1970 compared with cancer incidence in geographical control group. Persons aged 1-39 years. Number of cancer cases in Thyborøn-Harboøre (N), rate ratio (RR), and 95% confidence interval (CI). Blank cell means cancer site not relevant in that sex, or less than 5 observed cases

| Cancer site | Total | | | Men | | | Women | | |
|---|---|---|---|---|---|---|---|---|---|
| | N | RR | 95% CI | N | RR | 95% CI | N | RR | 95% CI |
| All cancer | 37 | 0.99 | 0.66-1.47 | 16 | 1.06 | 0.57-1.95 | 21 | 0.94 | 0.56-1.59 |
| All cancer, excl. other skin | 36 | 0.96 | 0.64-1.44 | 15 | 0.99 | 0.53-1.86 | 21 | 0.94 | 0.56-1.59 |
| Malignant melanoma | 5 | 1.36 | 0.43-4.29 | | | | | | |
| Leukemia | 6 | 3.76 | 0.94-15.02 | | | | | | |

Supplementary Table 2. Cancer incidence for persons in Thyborøn-Harboøre in 1968-1970 compared with cancer incidence in geographical control group. Person aged 40-59 years. Number of cancer cases in Thyborøn-Harboøre (N), rate ratio (RR), and 95% confidence interval (CI). Blank cell means cancer site not relevant in that sex, or less than 5 observed cases

| Cancer site | Total | | | Men | | | Women | | |
|---|---|---|---|---|---|---|---|---|---|
| | N | RR | 95% CI | N | RR | 95% CI | N | RR | 95% CI |
| All cancer | 300 | 1.23 | 1.07-1.43 | 139 | 1.37 | 1.10-1.70 | 161 | 1.14 | 0.93-1.38 |
| All cancer, excl. other skin | 297 | 1.28 | 1.10-1.48 | 137 | 1.43 | 1.15-1.79 | 160 | 1.17 | 0.96-1.42 |
| Stomach | 12 | 7.24 | 2.04-25.65 | 11 | 9.89 | 2.19-44.63 | | | |
| Colon and rectum | 38 | 1.36 | 0.90-2.07 | 23 | 1.17 | 0.97-3.01 | 15 | 1.03 | 0.55-1.94 |
| Colon | 16 | 0.87 | 0.48-1.58 | 11 | 1.27 | 0.59-2.74 | 5 | 0.51 | 0.19-1.39 |
| Rectum | 23 | 2.39 | 1.29-4.42 | 13 | 2.67 | 1.14-6.26 | 10 | 2.07 | 0.84-5.09 |
| Larynx | 7 | 2.61 | 0.83-8.24 | 6 | 2.24 | 0.68-7.34 | | | |
| Lung | 35 | 1.28 | 0.83-1.96 | 21 | 1.44 | 0.82-2.56 | 14 | 1.08 | 0.56-2.09 |
| Breast | 66 | 1.37 | 1.00-1.88 | | | | 65 | 1.35 | 0.98-1.86 |
| Cervix uteri | 9 | 1.04 | 0.46-2.36 | | | | 9 | 1.04 | 0.46-2.36 |
| Corpus uterus | 8 | 0.99 | 0.42-2.34 | | | | 8 | 0.99 | 0.42-2.34 |
| Ovary | 10 | 1.16 | 0.52-2.55 | | | | 10 | 1.16 | 0.52-2.55 |
| Prostate | 6 | 0.67 | 0.26-1.69 | 6 | 0.67 | 0.26-1.69 | | | |
| Kidney | 12 | 2.21 | 0.95-5.11 | 8 | 1.83 | 0.69-4.88 | | | |

| | | | | | | | | | |
|---|---|---|---|---|---|---|---|---|---|
| Bladder and urinary tract | 26 | 1.92 | 1.11-3.33 | 20 | 2.62 | 1.32-5.19 | 6 | 1.02 | 0.38-2.75 |
| Malignant melanoma | 15 | 1.28 | 0.67-2.47 | 5 | 1.17 | 0.38-3.57 | 10 | 1.34 | 0.60-3.02 |
| Other skin | 5 | 0.47 | 0.18-1.25 | | | | | | |
| Brain and CNS | 15 | 1.32 | 0.68-2.57 | 7 | 0.99 | 0.40-2.49 | 8 | 1.86 | 0.70-4.96 |
| Non-Hodgkin lymphoma | 11 | 1.35 | 0.62-2.94 | 7 | 1.62 | 0.59-4.47 | | | |
| Leukemia | 7 | 1.89 | 0.66-5.38 | 6 | 2.89 | 0.81-10.24 | | | |
| Other specified | | | | | | | | | |
| Unknown and unspecified | 10 | 1.24 | 0.56-2.75 | 5 | 1.04 | 0.35-3.10 | 5 | 1.55 | 0.47-5.09 |

Supplementary Table 3. Cancer incidence for persons in Thyborøn-Harboøre in 1968-1970 compared with cancer incidence in geographical control group. Person aged 60+ years. Number of cancer cases in Thyborøn-Harboøre (N), rate ratio (RR), and 95% confidence interval (CI). Blank cell means cancer site not relevant in that sex, or less than 5 observed cases

| Cancer site | Total | | | Men | | | Women | | |
|---|---|---|---|---|---|---|---|---|---|
| | N | RR | 95% CI | N | RR | 95% CI | N | RR | 95% CI |
| All cancer | 771 | 1.19 | 1.09-1.30 | 444 | 1.14 | 1.02-1.29 | 327 | 1.26 | 1.09-1.44 |
| All cancer, excl. other skin | 740 | 1.20 | 1.09-1.31 | 424 | 1.16 | 1.03-1.31 | 316 | 1.25 | 1.09-1.44 |
| Oral cavity | 5 | 0.98 | 0.33-2.87 | | | | | | |
| Oropharynx | 5 | 1.63 | 0.50-5.34 | | | | | | |
| Oesophagus | 15 | 1.15 | 0.61-2.18 | 12 | 1.10 | 0.54-2.23 | | | |
| Stomach | 27 | 1.30 | 0.81-2.09 | 20 | 1.32 | 0.76-2.30 | 7 | 1.25 | 0.50-3.17 |
| Small bowel | 6 | 5.70 | 1.15-28.26 | | | | | | |
| Colon and rectum | 147 | 1.35 | 1.10-1.67 | 65 | 1.01 | 0.75-1.36 | 82 | 1.85 | 1.37-2.49 |
| Colon | 95 | 1.36 | 1.05-1.76 | 37 | 0.94 | 0.63-1.38 | 58 | 1.90 | 1.33-2.72 |
| Rectum | 56 | 1.38 | 0.98-1.94 | 28 | 1.13 | 0.71-1.79 | 28 | 1.77 | 1.07-2.95 |
| Primary liver | 9 | 1.01 | 0.45-2.24 | | | | 6 | 2.08 | 0.67-6.47 |
| Pancreas | 28 | 1.50 | 0.92-2.45 | 17 | 1.87 | 0.97-3.61 | 11 | 1.16 | 0.55-2.44 |
| Larynx | 13 | 1.70 | 0.81-3.58 | 11 | 1.56 | 0.71-3.43 | | | |
| Lung | 514 | 1.38 | 1.11-1.70 | 98 | 1.54 | 1.18-2.01 | 47 | 1.13 | 0.78-1.61 |
| Breast | 74 | 1.14 | 0.86-1.52 | | | | 72 | 1.12 | 0.84-1.49 |
| Cervix uteri | 7 | 2.33 | 0.78-6.94 | | | | 7 | 2.33 | 0.78-6.94 |
| Corpus uterus | 20 | 1.56 | 0.87-2.79 | | | | | | |
| Ovary | 15 | 1.55 | 0.79-3.06 | | | | 15 | 1.55 | 0.79-3.06 |

| | | | | | | | | | |
|---|---|---|---|---|---|---|---|---|---|
| Prostate | 101 | 1.02 | 0.80-1.29 | 101 | 1.02 | 0.80-1.29 | | | |
| Kidney | 24 | 1.78 | 1.03-3.07 | 16 | 1.63 | 0.84-3.16 | 8 | 2.11 | 0.79-5.64 |
| Bladder and urinary tract | 80 | 1.76 | 1.31-2.37 | 61 | 1.59 | 1.14-2.21 | 10 | 2.78 | 1.39-5.55 |
| Malignant melanoma | 23 | 1.37 | 0.80-2.35 | 9 | 2.42 | 0.90-6.49 | 14 | 1.08 | 0.56-2.07 |
| Other skin | 50 | 1.18 | 0.83-1.67 | 33 | 1.17 | 0.76-1.80 | 17 | 1.20 | 0.66-2.18 |
| Brain and CNS | 17 | 0.99 | 0.55-1.78 | 6 | 0.71 | 0.28-1.82 | 11 | 1.26 | 0.59-2.69 |
| Non-Hodgkin lymphoma | 19 | 0.92 | 0.53-1.59 | 9 | 0.87 | 0.40-1.90 | 10 | 0.96 | 0.45-2.06 |
| Myelomatosis | 6 | 0.68 | 0.27-1.72 | | | | | | |
| Leukemia | 17 | 0.98 | 0.55-1.74 | 13 | 1.14 | 0.58-2.24 | | | |
| Other specified | 5 | 3.31 | 0.79-13.89 | | | | | | |
| Unknown and unspecified | 41 | 1.11 | 0.76-1.62 | 22 | 1.08 | 0.65-1.80 | 19 | 1.15 | 0.65-2.01 |

Supplementary Table 4. Cancer incidence for persons in Thyborøn-Harboøre in 1990-2006 compared with cancer incidence in persons in Thyborøn-Harboøre in 1968-1970. Number of cancer cases in Thyborøn-Harboøre in 1990-2006 (N), rate ratio (RR), and 95% confidence interval (CI). Only cancer sites with excess cancer incidence in Thyberøn-Harboøre in 1968-1970 as compared with the geographical control group and with 5+ observed cancer cases in the 1990-2006 Thyborøn-Harboøre cohort. Blank cell means cancer site not relevant in that sex, or less than 5 observed cases

| Cancer site | Total | | | Men | | | Women | | |
|---|---|---|---|---|---|---|---|---|---|
| | N | RR | 95% CI | N | RR | 95% CI | N | RR | 95% CI |
| All cancer | 126 | 1.00 | 0.82-1.21 | 64 | 0.89 | 0.67-1.17 | 62 | 1.15 | 0.86-1.52 |
| Colon and rectum | 14 | 0.75 | 0.43-1.32 | 8 | 0.73 | 0.35-1.53 | 6 | 0.76 | 0.32-1.80 |
| Colon | 7 | 0.60 | 0.27-1.31 | | | | | | |
| Rectum | 7 | 0.90 | 0.40-2.00 | 5 | 1.00 | 0.38-2.60 | | | |
| Lung | 23 | 1.19 | 0.75-1.87 | 10 | 0.81 | 0.41-1.57 | 13 | 1.88 | 1.00-3.54 |
| Kidney | 5 | 1.53 | 0.55-4.24 | | | | | | |
| Bladder and urinary tract | 11 | 1.02 | 0.53-1.95 | 7 | 0.84 | 0.38-1.87 | | | |
